# Distinct Yet Coordinated: Pattern of Leaf and Root Fungal Endophyte Communities in an Alpine Meadow

**DOI:** 10.3390/jof12080580

**Published:** 2026-08-07

**Authors:** Miao Dong, Xiaoli Hu, Shuaibing He

**Affiliations:** Department of Biology, School of Life Sciences, Nanjing University, Nanjing 210023, China; biodiv_hu@nju.edu.cn (X.H.); 15670523502@163.com (S.H.)

**Keywords:** leaf fungal endophyte, root fungal endophyte, community divergence, coordinated assembly

## Abstract

Leaf and root fungal endophytes (LFEs and RFEs) represent essential yet distinct components of the plant holobiont, but their ecological relationships remain poorly understood. Here, we investigated the community structure of LFE and RFE across 45 plant species in an alpine meadow ecosystem using high-throughput sequencing. We found that the richness of RFEs was significantly higher than that of LFEs whereas their diversity showed no significant difference. The community composition of RFEs significantly differed with LFE communities and indicator analysis revealed that *Aureobasidium*, *Didymella*, and *Mycosphaerella* enriched in leaves and *Mucor*, *Candida*, and *Trichoderma* dominating roots. Fungal endophytes form a complementary functional network that supports plant growth, defense, and resource utilization. Plant functional traits rather than phylogenetic distance shaped the structure of both LFE and RFE communities. Despite their compositional divergence, LFEs and RFEs shared a large number of taxa and showed a coordinated assembly pattern. The coordinated variation between leaf and root functional traits, coupled with the fluxes of LFEs and RFEs, is a critical driver of this pattern. Overall, our results provide integrated evidence that LFE and RFE communities constitute ecologically distinct yet interconnected components of the plant holobiont, jointly contributing to plant performance and ecosystem functioning.

## 1. Introduction

Plant fungal endophytes are ubiquitous components of terrestrial ecosystems and play essential roles in host growth, stress tolerance, and defense [1]. These fungi inhabit internal plant tissues without causing apparent disease symptoms [2] and establish intricate interactions that can range from mutualism to latent pathogenicity [3,4]. Over the past decades, increasing attention has been paid to the ecological diversity and functional significance of fungal endophytes in natural ecosystems, as they contribute to plant adaptation under environmental stress [5,6,7].

Leaves and roots represent two highly contrasting microhabitats: although they are capable of providing nutrients to fungal endophytes, the phyllosphere is characterized by exposure to fluctuating temperature, desiccation, and UV radiation [8], whereas the rhizosphere provides a nutrient-rich and stable environment [9]. Such differences are expected to drive strong compositional divergence between foliar and root fungal endophyte communities. For instance, the diversity of endophytic fungi was greater in the roots than in the leaves of *Hevea brasiliensishe* native to the Brazilian Amazon forest [10] and the diversity of endophytic fungi was significantly higher in roots as compared to leaves of *Stipa purpurea* in the Qinghai–Tibet Plateau [11]. Meanwhile, the endophytic fungi of *Cinnamomum longepaniculatum* showed higher richness and diversity in leaves than in roots [12].

Despite this growing body of research, most studies have focused on distribution of either leaf or root endophyte communities in isolation, while the linkages between leaf and root fungal endophytes remain poorly understood. Previous studies showed that both plant phylogeny and functional traits could affect fungal endophyte communities. For instance, Liu et al. [13] demonstrated that both host identity and phylogeny drive foliar endophytic fungal assemblages in *Ficus* within Xishuangbanna. Similarly, leaf resistance traits have been shown to influence the colonization and community composition of leaf endophytes in South American temperate rainforests [14]. Beyond above-ground tissues, host plant identity also dictates the colonization patterns and species composition of root fungal endophytes, as evidenced by studies on medicinal plants in northern Chinese farmlands [15] and three plant species in the Mu Us sandy land of northwest China [16]. Because a plant is an integrated biological system, relations exist between root and leaf traits [17], and phylogeny also affects plant functional traits [18]. Thus, fungal endophyte communities inhabiting leaves and roots may not be entirely independent.

In alpine ecosystems, where plants experience strong abiotic stress and limited growing seasons [19], endophytic fungi are particularly important in enhancing host adaptability. Yet, whether and how above- and belowground fungal endophyte communities are interrelated in such extreme environments remains largely unexplored. Here, we conducted a paired analysis of foliar and root fungal endophyte communities across 45 alpine plant species. Specifically, we asked: (1) Do foliar and root fungal endophyte communities differ in abundance and diversity? (2) Are abundance, diversity, and functional guilds of foliar and root fungal endophytes correlated across plant species? and (3) Are these communities shaped by host phylogeny and functional traits? By addressing these questions, this study aims to elucidate the degree of ecological coupling between above- and belowground fungal endophytes and to provide new insights into the integrative nature of plant–microbe associations in alpine ecosystems.

## 2. Materials and Methods

### 2.1. Study Site

The study site is an alpine meadow (~3500 m ASL) in Hongyuan County, Sichuan Province, China, situated on the eastern Tibetan Plateau (32°83′ N, 102°58′ E). The area has a mean annual temperature of 1.7 °C (January: −9.3 °C; July: 11.1 °C) and receives a mean annual precipitation of 756 mm (450–900 mm), mostly between May and September. Yaks (*Bos grunniens*) graze the meadow seasonally during the non-growing season. The vegetation exhibits high coverage (>90%) and species richness (25–30 species m^−2^), dominated by forbs (*Saussurea nigrescens*, *Anaphalis flavescens*, *Potentilla discolor*, *Polygonum viviparum*), grasses (*Deschampsia caespitosa*, *Festuca rubra*, *Elymus nutans*), and legumes (*Lathyrus quinquenervius*, *Hedysarum sikkimense*) [20].

### 2.2. Root and Leaf Traits and Species Abundance

In mid-July 2020, we collected leaf and root samples from 45 healthy plant species (no visible symptoms of disease or herbivory were observed) (Table S1) within a 100 × 150 m area. Plant samples were collected by excavating five individuals for each target species. To preserve the integrity of root systems, large soil blocks exceeding the estimated root spread were extracted using a turf shovel. To obtain intact root systems, each soil block was submerged in water for several hours to loosen the substrate. The roots were then gently rinsed with low-pressure water until all adhering soil particles were completely dislodged.

The total root length (from the soil surface to the deepest root tip) was measured with a ruler. After removing surface moisture by blotting with absorbent paper, roots were weighed to obtain fresh weight, then oven-dried at 75 °C for 48 h to determine dry weight. Root water content (WC) was calculated as the difference between fresh and dry weights, expressed as a proportion of fresh weight.

To assess leaf morphological traits, leaf area was digitized using a Canon LiDE 300 scanner (Canon, Tokyo, Japan) and processed in ImageJ (version 1.53c; National Institutes of Health, Bethesda, MD, USA). Leaves were subsequently dried at 75 °C for 48 h to determine dry weight, allowing for the calculation of leaf mass per area (LMA) based on the dry weight-to-area ratio. Leaf water content was assessed using a method identical to that applied to roots. For chemical trait analysis, carbon (C) and nitrogen (N) concentrations in both roots and leaves were quantified via an elemental analyzer (Vario EL III, Elementar, Langenselbold, Germany).

### 2.3. Root and Leaf Fungal Endophyte

For the 45 plant species, three biological replicates were used for DNA extraction of root and leaf fungal endophytes. Each replicate comprised approximately 0.8 g of fresh tissue, pooled from five individual plants distinct from those used for plant trait measurements. Prior to DNA extraction, samples were surface-sterilized sequentially in 75% ethanol for 2 min and 0.5% NaClO for 10 min, followed by three rinses with sterile distilled water. Total genomic DNA was then extracted using a Tiangen Plant DNA Kit (Tiangen, Beijing, China).

The fungal ITS2 region was amplified using the ITS4-Fun (5′-AGCCTCCGCTTATTGATATGCTTAART-3′) and gITS7F (5′-GTGARTCATCGARTCTTTG-3′). PCR amplification was performed in a 25 µL reaction volume comprising 12.5 µL of 2× Taq Master Mix (TsingKe Biological Technology Co., Ltd., Beijing, China), 1 µL each of forward and reverse primers, 9.5 µL of double-distilled water (ddH_2_O), and 1 µL of DNA template (10 ng/µL). The thermal cycling program consisted of an initial denaturation at 94 °C for 5 min, followed by 34 cycles of denaturation at 94 °C for 30 s, annealing at 56 °C for 30 s, and extension at 68 °C for 45 s, with a final extension at 72 °C for 10 min. Amplicon sequencing was performed on an Illumina MiSeq platform (2 × 250 bp paired-end reads; Illumina, San Diego, CA, USA) at Biobert Biotechnologies Co., Ltd. (Chengdu, China). The resulting raw sequences were processed and clustered into operational taxonomic units (OTUs) at a 97% similarity threshold using QIIME v1.9.1. Subsequently, fungal functional guilds were annotated based on the FUNGuild database (v1.1) [21].

### 2.4. The Marker OTUs of RFEs and LFEs

To identify marker OTUs associated with both RFEs and LFEs, we employed specificity and occupancy metrics. Following the framework established by Dufrêne and Legendre [22], specificity is defined as the mean relative abundance of a given OTU across samples within a specific plant organ, while occupancy represents the proportion of samples in which that OTU is detected. The calculations for these two metrics were performed as follows:

$$Specificity=\frac{{Nindividuals}_{S,H}}{{Nindividuals}_{S}}$$

$$Occupancy=\frac{{Nsites}_{S,H}}{{Nsites}_{S}}$$

Here, Nindividuals_S,H_ represents the mean abundance of a specific OTU across all samples within a given organ, while Nindividuals_S_ denotes the sum of these mean abundances across all organs. Similarly, Nsites_S,H_ indicates the number of samples in which the OTU is present within that organ, and Nsites_S_ refers to the total number of samples for that organ. Following the criteria proposed by Gweon [23], OTUs with both specificity and occupancy values ≥ 0.7 were identified as marker OTUs for their respective plant organs.

### 2.5. Statistical Analysis

We utilized the diversity function in the R package vegan v. 2.5-4 [24] to compute OTU richness and Shannon diversity indices. Subsequently, generalized linear models (GLMs), executed via the ‘glm’ function in the same package, were employed to examine potential differences in fungal endophyte diversity and richness between root and leaf tissues. The relationships of the richness and Shannon diversity between RFE and LFE were also assessed using GLMs. Furthermore, GLMs were applied to evaluate the relationships of abundances of endophytic fungi with different trophic modes between roots and leaves. Fungal abundance data were log-transformed prior to analysis to minimize the influence of outliers.

The plant phylogenetic tree was retrieved from the Open Tree of Life mega-tree using the ‘get_tree’ function in the R package rtrees v. 1.0.3 [25]. Subsequently, pairwise phylogenetic distances among the plant species were computed based on this tree via the ‘cophenetic’ function in the R package phangorn v. 2.11.1 [26]. Additionally, the Bray–Curtis dissimilarity between each pair of plant species for both RFEs and LFEs was calculated via the ‘vegdist’ function in vegan v. 2.5-4. The Mantel test with 999 permutations was used to assess (1) the correlations between plant phylogenetic distances and community dissimilarities of RFEs and LFEs, respectively, and (2) the correlation between RFE and LFE community dissimilarities.

To visualize the beta diversity of RFEs and LFEs, Principal Coordinates Analysis (PCoA) was performed based on Bray–Curtis distances. Furthermore, to determine whether the endophyte community compositions differed significantly between root and leaf tissues, a permutational multivariate analysis of variance (PERMANOVA) was conducted using the ‘adonis2’ function in vegan v. 2.5-4. In addition, Mantel tests with 999 permutations were used to examine the correlations between Bray–Curtis dissimilarities of RFE/LFE communities and Euclidean distance matrices of individual plant trait variables. The relationships among plant functional traits were assessed using the ‘corr.test’ function in the R package psych v. 2.2.5 [27].

## 3. Results

**Figure 1:**
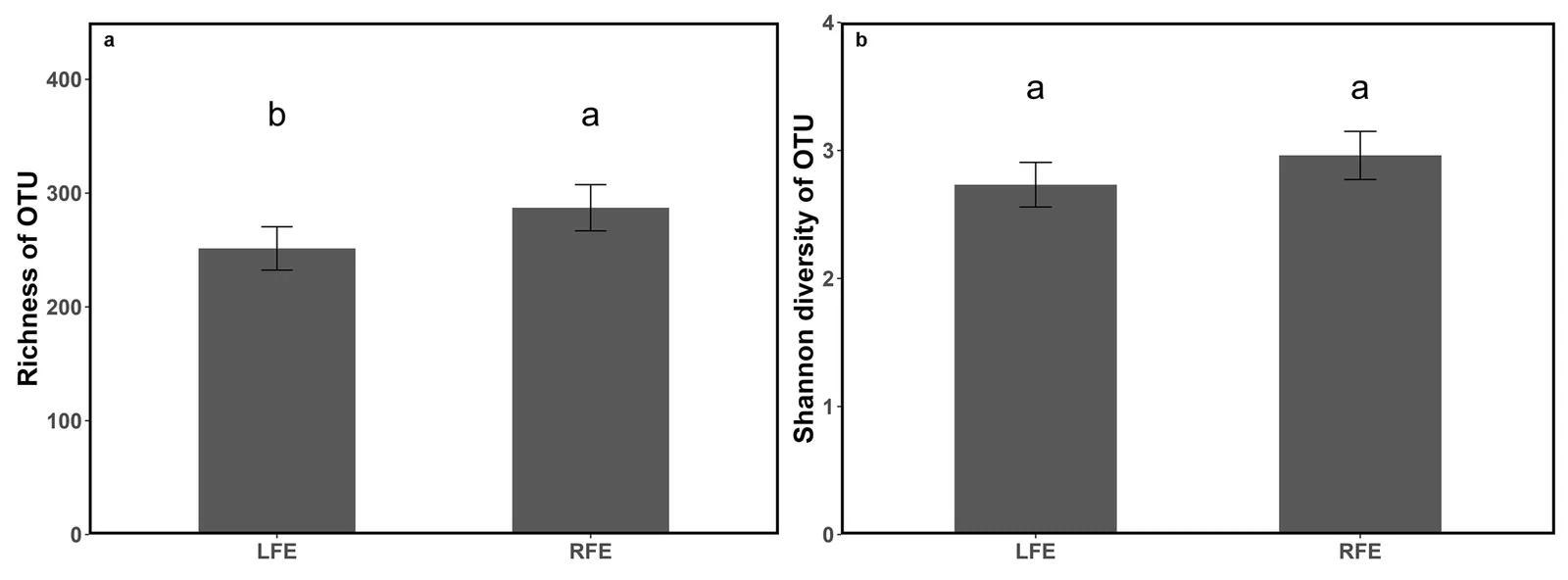
Significant variation in fungal endophyte alpha diversity between leaves and roots. Difference in richness (**a**) and Shannon diversity (**b**) of OTU between leaves and roots. Different letters above the bars denote statistically significant differences between leaves and roots at the level of *p* < 0.05.

**Figure 2:**
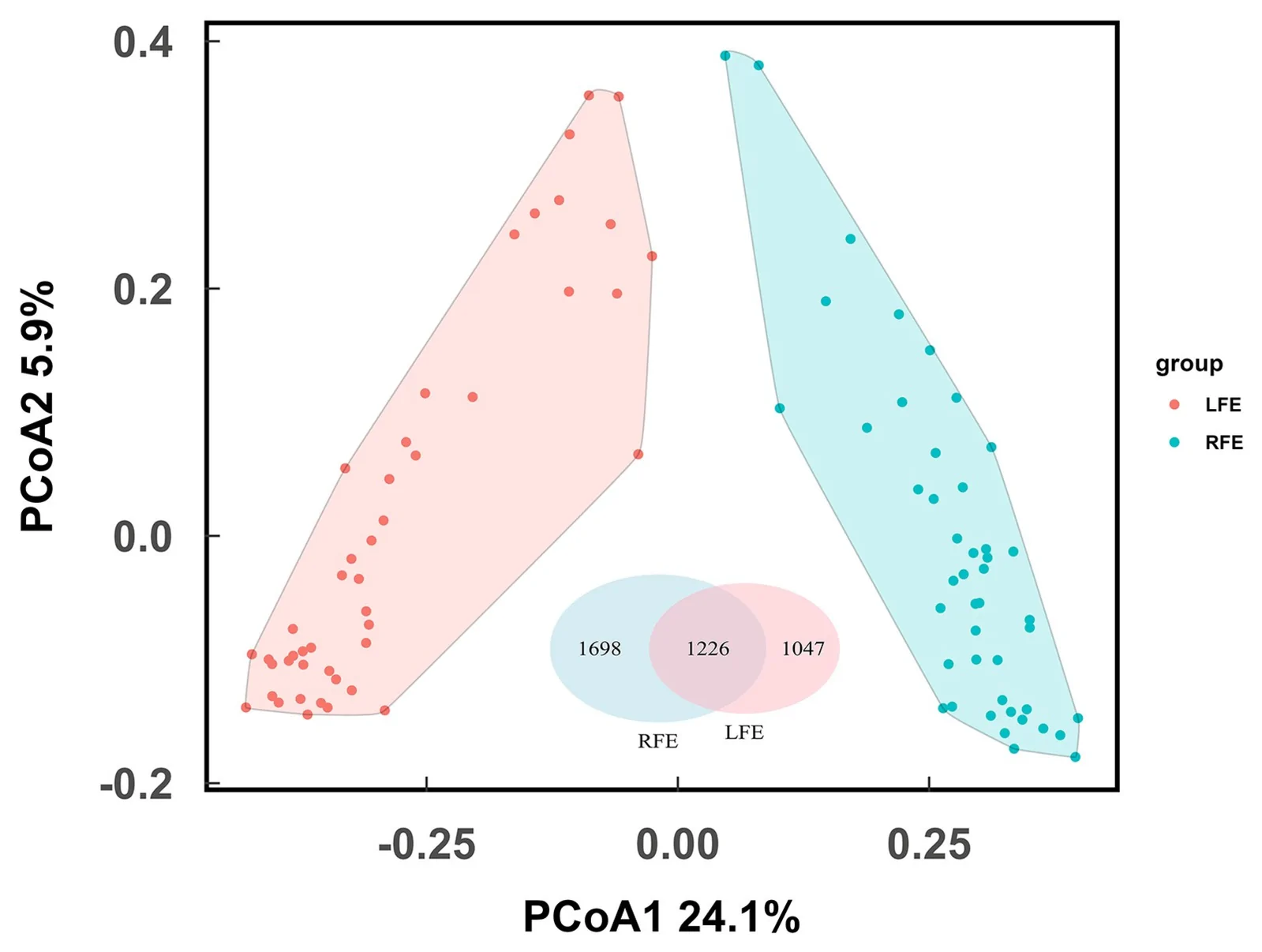
Beta diversity and shared OTUs of fungal endophyte communities in leaves and roots. PCoA plot of the fungal composition based on Bray–Curtis distances at the OTU level between leaves and roots. The Venn diagram showing the number of shared and unique OTUs between leaves and roots.

**Figure 3:**
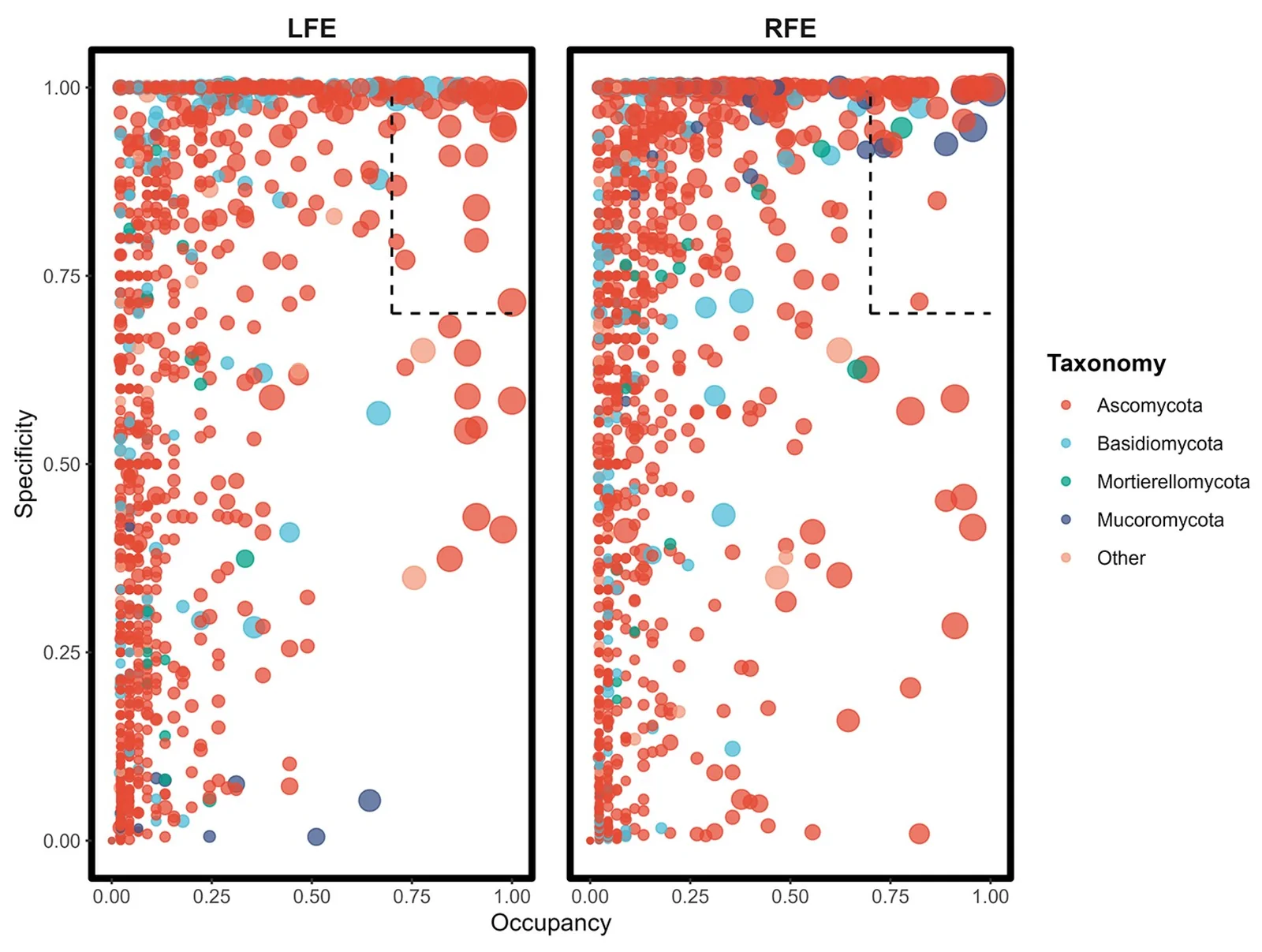
The SPEC-OCCU plots show marker OTUs in leaves and roots. The x-axis represents occupancy and the y-axis represents specificity. The points within the dashed box are the marker OTUs.

The richness of LFEs was significantly lower than that of RFEs (Figure 1a), whereas no significant difference was observed in Shannon diversity between LFE and RFE communities (Figure 1b). The result of PERMANOVA shows that the community composition of RFE and LFE differed significantly (*p* < 0.05). A total of 1226 OTUs were shared between roots and leaves, while 1698 OTUs were unique to roots and 1047 OTUs were unique to leaves (Figure 2). A total of 44 marker OTUs were identified for LFE and 38 marker OTUs for RFE. Only one marker OTU from both compartments belonged to the same genus, while all other marker OTUs were affiliated with distinct genera. The dominant genera among LFE marker OTUs were *Mycosphaerella*, *Aureobasidium* and *Didymella*, whereas the dominant genera among RFE marker OTUs were *Mucor*, *Candida* and *Trichoderma* (Figure 3).

**Figure 4:**
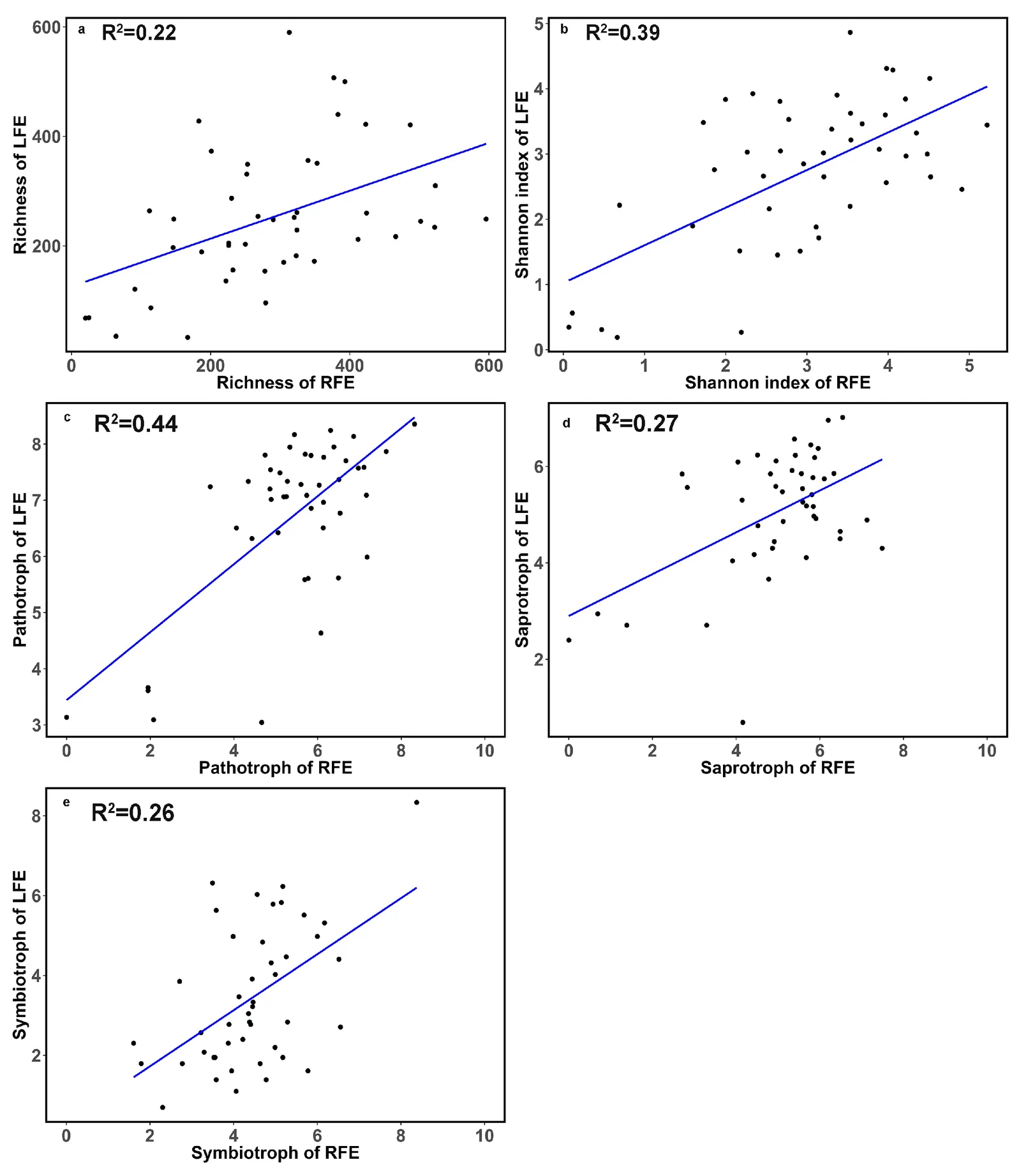
Leaf-root correlations in fungal endophyte alpha diversity and trophic mode abundance. Correlation of richness (**a**) and Shannon diversity (**b**) of OTU between leaves and roots. Correlation of abundance of pathogens (**c**), saprotrophs (**d**) and symbionts (**e**) between leaves and roots. Fungal abundance data of pathogens, saprotrophs and symbionts were log-transformed. Blue line denotes statistically significant correlation between leaves and roots at the level of *p* < 0.05. Each black dot represents a plant species.

The richness of LFE was significantly positively correlated with that of RFE (Figure 4a), and a similar positive correlation was found between their Shannon diversity indices (Figure 4b). Both LFE and RFE richness showed a significantly positive correlation with the richness of their shared OTUs (Figure S1a,b). The abundances (log-transformed) of pathogenic, saprotrophic, and symbiotic fungi in leaves were all significantly positively correlated with the corresponding abundances (log-transformed) of pathogenic, saprotrophic, and symbiotic fungi in roots (Figure 4c–e).

**Figure 5:**
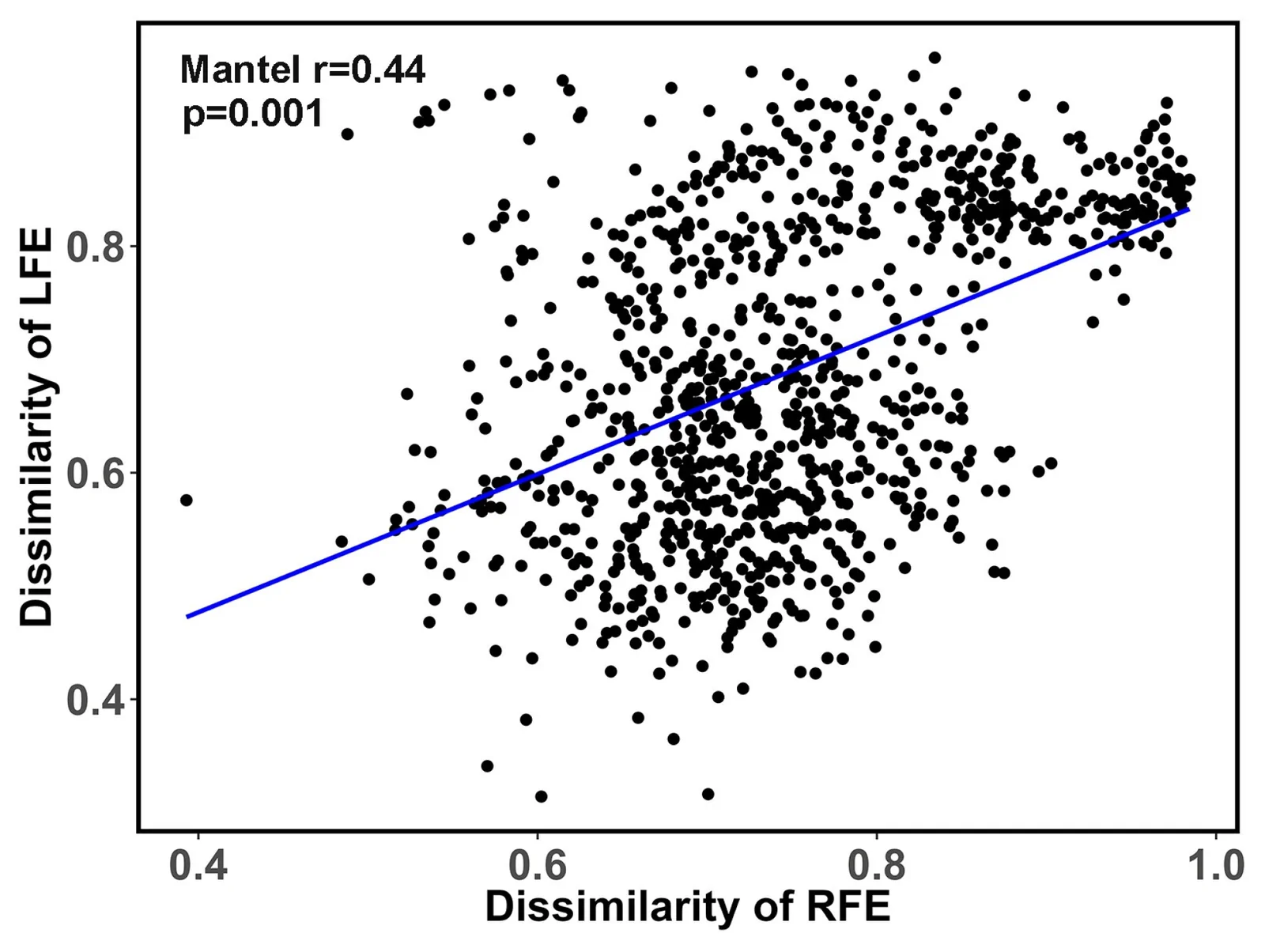
Correlation of fungal community composition between LFE and RFE. Mantel test between the LFE and RFE composition based on Bray–Curtis distances at the OTU level. Blue line denotes statistically significant correlation between leaves and roots at the level of *p* < 0.05. Each point represents a pair of plants.

The compositional dissimilarity of RFE communities was not significantly correlated with plant phylogenetic distance (Figure S2), and a similar lack of correlation was observed for LFE communities (Figure S3). However, the compositional dissimilarities of leaf and root endophytic fungal communities were significantly positively correlated (Figure 5).

**Figure 6:**
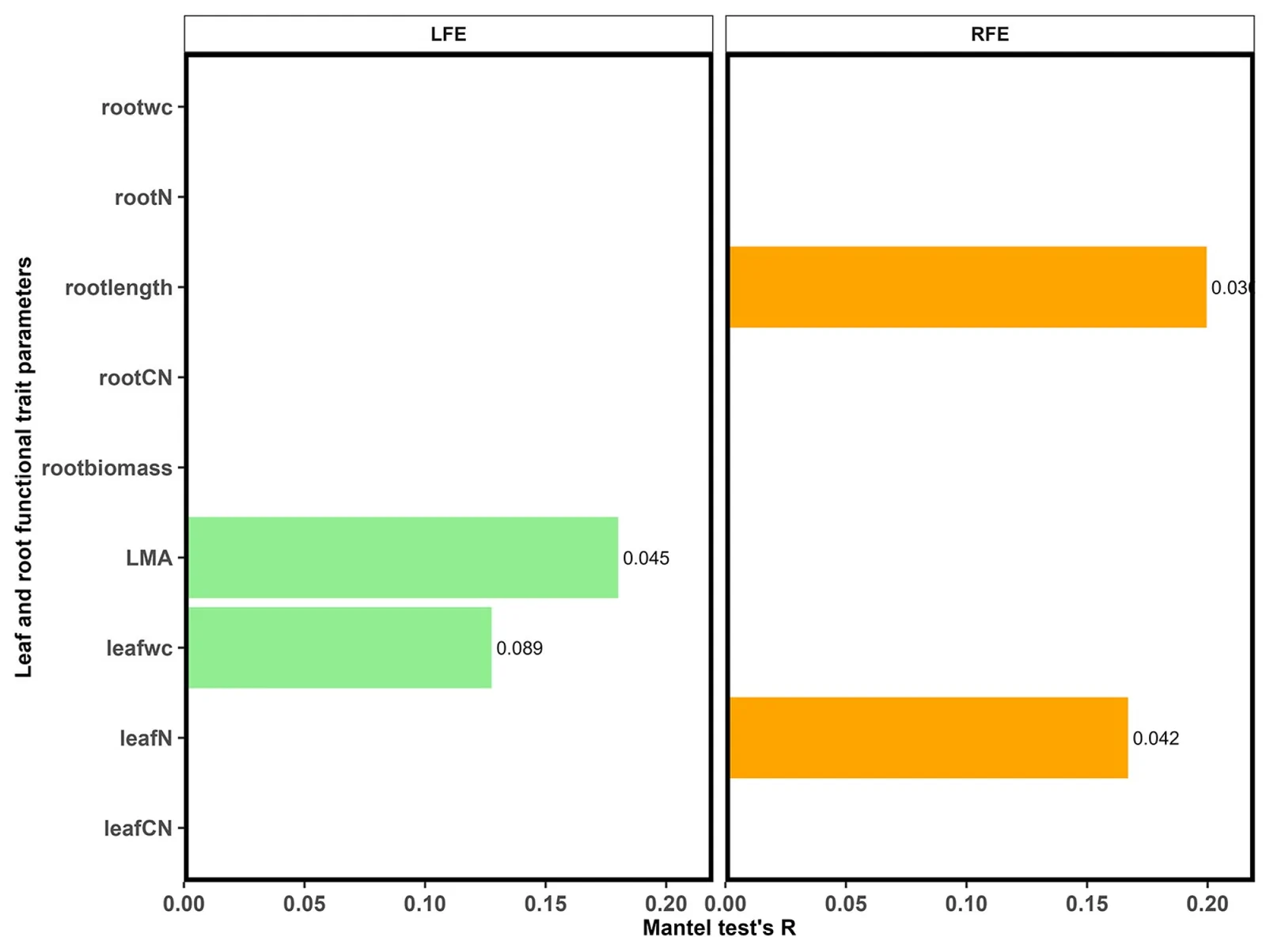
Associations between plant traits and fungal endophyte communities in leaves and roots. Mantel tests between each plant trait variable (Euclidian distance matrix) and LFE/RFE community structures (Bray–Curtis distance matrices), the numbers are the *p* values. Green represents the LFE, and orange represents the RFE. Rootwc: root water content, rootN: root nitrogen concentration, rootCN: root carbon and nitrogen ratio, LMA: leaf mass per area, leafwc: leaf water content, leafN: leaf nitrogen concentration, leaf CN: leaf carbon and nitrogen ratio.

Mantel tests revealed correlations between various plant trait variables and the community structures of both LFE and RFE. Specifically, the composition of LFE communities was significantly correlated with leaf mass per area (LMA) and showed a marginally significant correlation with leaf water content, whereas the composition of RFE communities was significantly correlated with root length and leaf nitrogen concentration. Other traits such as root water content, root nitrogen concentration and root biomass did not exhibit significant effect on the community structures of either LFEs or RFEs (Figure 6). Significant correlations were detected among nine pairs of plant functional traits (Figure S4).

## 4. Discussion

Our study provides integrated evidence that leaf and root fungal endophyte (LFE and RFE) communities are tightly coupled but ecologically distinct components of the plant holobiont. Despite the significant compositional differences between the two communities, a large number of shared taxa were still detected, and a coordinated assembly pattern was observed between them.

Our study found that RFEs exhibited significantly higher OTU richness than LFEs. This may be attributed to the abundance of litter residues (such as fallen leaves, twigs, and dead roots) at the soil–root interface, which supply essential nutrient elements (such as carbon and nitrogen) to stimulate the proliferation of root-associated fungi [28]. In contrast, microbial colonization in leaves typically begins with epiphytic settlement on the leaf surface through stomata, hydathodes, or wounds, followed by horizontal transmission and penetration into internal tissues [29]. However, exposure to ultraviolet radiation, nutrient limitation, and desiccation in the aboveground environment create unfavorable conditions for microbial growth [8], leading to lower fungal abundance in leaves. In addition, the longer annual persistence of roots in soil relative to leaves may also account for the greater abundance of endophytic fungi in roots [30]. These findings are consistent with previous studies on the endophytic fungal communities of *Mussaenda kwangtungensis* [31] and *Stipa purpurea* [11], which have shown the RFE had significantly higher OTU richness than LFE. Interestingly, our results further showed that the diversity of RFEs did not differ significantly from that of LFEs. This suggests that although the RFE community exhibits a higher overall richness, competition for resources leads to the dominance of a small number of highly competitive species, whereas the LFE community, despite its lower total richness, shows a more even distribution among taxa.

Although no significant difference in diversity was observed between LFEs and RFEs, their community compositions differed significantly. This pattern likely reflects the organ-specific preferences of fungal taxa within different plant tissues [32]. Aside from approximately 1000 shared OTUs, 1200 OTUs were unique to RFEs and 1300 OTUs were unique to LFEs. Such organ-specific differentiation in endophytic fungal communities has also been reported in *Salix* [33], *Betula* [34], Cactaceae [35] and *Agave* [36]. In this study, neither LFE nor RFE community composition was correlated with host phylogenetic distance, while plant functional traits play important roles in fungal community assembly. Specifically, LFE composition is significantly associated with leaf mass per area (LMA). LMA reflects leaf thickness and tissue density, thereby shaping plant resource allocation strategies. Plants exhibiting low LMA generally produce thin leaves with lower tissues density and reduced physical barriers, representing a fast-growing strategy, with limited defensive investment [37]. This morphological profile effectively facilitates the colonization and dissemination of LFE [38], corroborating earlier research conducted on tropical tree communities [13,39]. The RFE composition is significantly associated with root length. The longer roots can reach deeper soil layers and have a greater contact area with the soil. One important origin of RFEs is soil [40]; therefore, plant with longer roots provide more potential opportunities for soil fungi to colonize into roots. Beyond their physical presence, roots actively release a diverse array of compounds collectively known as root exudates. These secretions include amino acids, organic acids, sugars, hormones, nucleotides, inorganic substances, peptides, polysaccharides, and proteins [41,42]. These metabolites function as critical drivers that shape both the composition and assembly of the RFEs.

Further analysis of marker OTUs revealed dozens of marker OTUs for both LFE and RFE communities; however, only one OTU belonged to the same genus between the two compartments, while all others were affiliated with distinct genera. In the LFE community, *Mycosphaerella*, *Aureobasidium*, and *Didymella* were identified as dominant indicator taxa. Among them, *Aureobasidium* and *Didymella* have been reported to exhibit antagonistic activity against other pathogenic fungi [43,44], suggesting their potential roles in host defense. *Mycosphaerella*, in contrast, has been recognized as a key network node in leaf decomposition systems [45], highlighting its importance in the turnover of foliar organic matter. In the root endophytic fungal community, *Mucor*, *Candida*, and *Trichoderma* were identified as dominant indicator taxa. *Mucor* is known to produce acid phosphatase [46], thereby facilitating plant phosphorus acquisition. *Candida* can synthesize sugar alcohols [47] that enhance plant metabolic activity [48], while *Trichoderma* promotes nutrient uptake and increases the concentrations of copper (Cu), iron (Fe), zinc (Zn), manganese (Mn), sodium (Na), and phosphorus (P) in plant roots [49] and has been widely used in plant bio protection [50]. Together, these findings suggest that endophytic fungi in leaves and roots form a complementary functional network that supports plant growth, defense, and resource utilization.

Although the diversity of LFE and RFE exhibits statistically significant differences, their diversity shows significant correlation in interspecific variation. Specifically, plant species with higher RFE richness tend to have higher LFE richness; plant species with higher RFE Shannon diversity tend to have higher LFE Shannon diversity; and plant species with more similar RFE community compositions tend to have more similar LFE community compositions. Additionally, the abundances of trophic guilds (pathogens, saprotrophs and symbionts) of LFE and RFE are significantly positively correlated across plant species. Previous studies have shown that fungal colonization in plant roots can induce systemic resistance (ISR), characterized by increased expression of pathogenesis-related (PR) genes, such as phenylalanine ammonia-lyase, polyphenol oxidase, peroxidase, β-1,3-glucanase, and chitinase, as well as the accumulation of reactive oxygen species [51,52]. These systemic defense responses can subsequently shape microbial colonization in aboveground tissues [53]. Therefore, in this study, plant species with more similar RFE communities may have triggered comparable ISR responses, thereby leading to more similar LFE community compositions. In addition, given that both LFE and RFE are strongly influenced by plant functional traits, and that these traits are highly coordinated between leaves and roots, such whole-plant trait coordination likely underpins the coupling between RFE and LFE communities. Finally, it has been reported that plant-associated microbial communities undergo progressive filtering from bulk soil to the rhizoplane, root interior, leaf surface, and ultimately the leaf interior [54]. In this study, both RFE and LFE richness were positively associated with the richness of shared OTUs, suggesting a potential linkage between belowground and aboveground communities. Such a pattern may help explain the coordinated variation observed between RFEs and LFEs. Overall, these results indicate that despite taxonomic differentiation, RFE and LFE communities are functionally and structurally coordinated, reflecting integrated host regulation and cross-compartment linkages in plant microbiome assembly.

## 5. Conclusions

LFE and RFE communities are ecologically distinct yet tightly interconnected components of the plant holobiont. Despite compositional differences, they share numerous taxa and exhibit coordinated assembly, reflecting strong ecological coupling. LFE primarily contributes to defense and leaf organic matter turnover, while RFE promotes nutrient acquisition and resource use. The coordinated variation between LFEs and RFEs is largely driven by covariation in plant functional traits, such as leaf mass per area and root length. Collectively, our study emphasizes the importance of considering both above- and belowground microbiomes to understand plant adaptation in alpine environments.

## Data Availability

Source data will be archived on Zenodo by the following link: https://doi.org/10.5281/zenodo.20539277.

## Supplementary Materials

The following supporting information can be downloaded at https://www.mdpi.com/article/10.3390/jof12080580/s1, Figure S1. Correlation between (a) LFE/(b) RFE richness and shared OTU richness. Blue line denotes statistically significant correlation between leaves and roots at the level of *p* < 0.05. Figure S2. Association between LFE composition and plant phylogenetic distance. Mantel test between the LFE composition based on Bray–Curtis distances and plant phylogentic distances at the OTU level. Figure S3. Association between RFE composition and plant phylogenetic distance. Mantel test between the RFE composition based on Bray–Curtis distances and plant phylogentic distances at the OTU level. Figure S4. Correlations of each plant functional traits. Numbers indicate correlation coefficients and only significant relationships (*p* < 0.05) are shown. LN: leaf nitrogen content; LCN: leaf carbon nitrogen ratio; LWC: leaf water content; LMA: leaf mass per area; RN: root nitrogen content; RCN: root carbon nitrogen ratio; RWC: root water content; RL: root length; RB: root biomass. Table S1. List of plant.

## Abbreviations

The following abbreviations are used in this manuscript:

LFE	Leaf fungal endophyte
RFE	Root fungal endophyte
PCoA	Principal coordinates analysis
OTU	Operational taxonomic units
GLM	Generalized linear model
LMA	Leaf mass per area
