# Supplementary material for: Distinct Yet Coordinated: Pattern of Leaf and Root Fungal Endophyte Communities in an Alpine Meadow
Source: J Fungi (Basel). 2026 Aug 7;12(8):580. doi: 10.3390/jof12080580 (PMC13514471; doi:10.3390/jof12080580)
Supplement: Supplementary file 1 [file jof-12-00580-s001.zip › jof-4424663-supplementary.pdf]

Supplementary information

Figure S1 Correlation between (a) LFE/ (b) RFE richness and shared OTU richness. Blue line denotes statistically significant correlation between leaves and roots at the level of $p < 0.05$.

Figure S2 Association between LFE composition and plant phylogenetic distance. Mantel test between the LFE composition based on bray-curtis distances and plant phylogentic distances at the OTU level.

Figure S3 Association between RFE composition and plant phylogenetic distance. Mantel test between the RFE composition based on bray-curtis distances and plant phylogentic distances at the OTU level.

Figure S4 Correlations of each plant functional traits. Numbers indicate correlation coefficients and only significant relationships ($p < 0.05$) are shown. LN: leaf nitrogen content; LCN: leaf carbon nitrogen ratio; LWC: leaf water content; LMA: leaf mass per area; RN: root nitrogen content; RCN: root carbon nitrogen ratio; RWC: root water content; RL: root length; RB: root biomass.

Table S1 List of plant

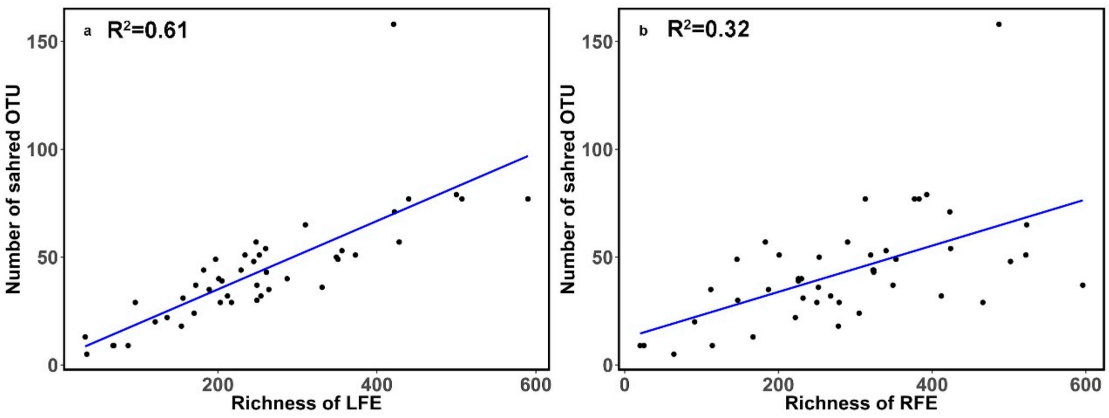
a
R^2=0.61
Number of sahred OTU
Richness of LFE
b
R^2=0.32
Number of sahred OTU
Richness of RFE
0
50
100
150
0
200
400
600

Figure S1

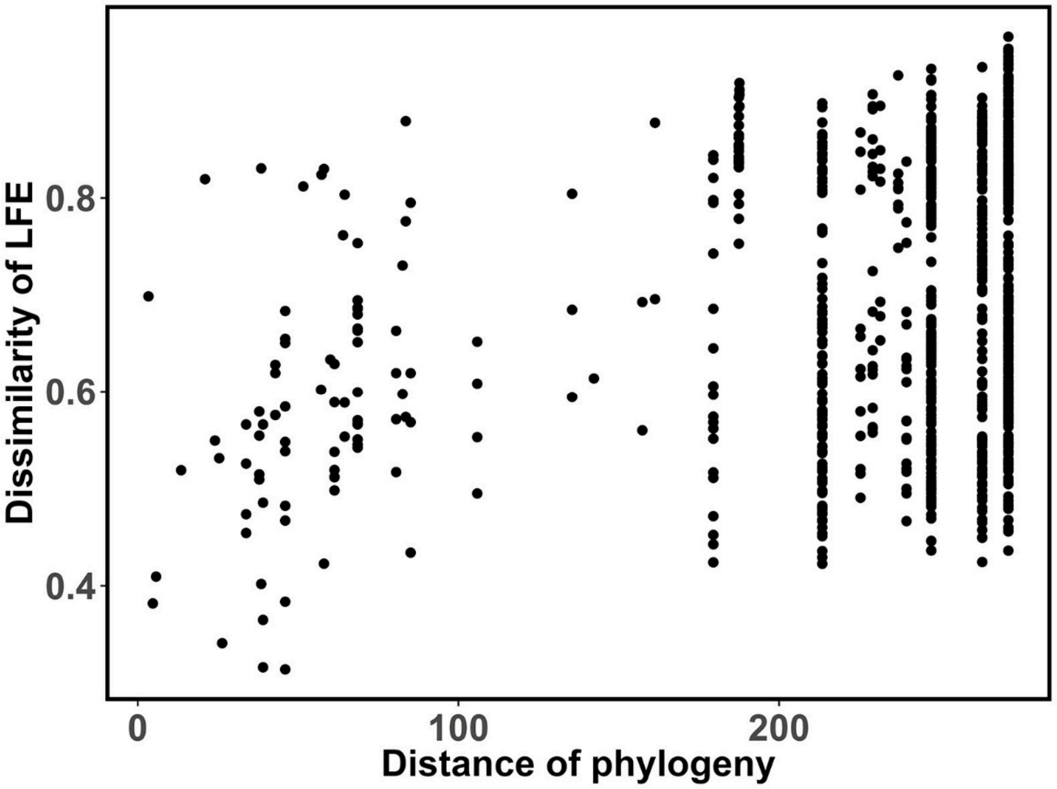
Dissimilarity of LFE
0.4
0.6
0.8
0
100
200
Distance of phylogeny

Figure S2

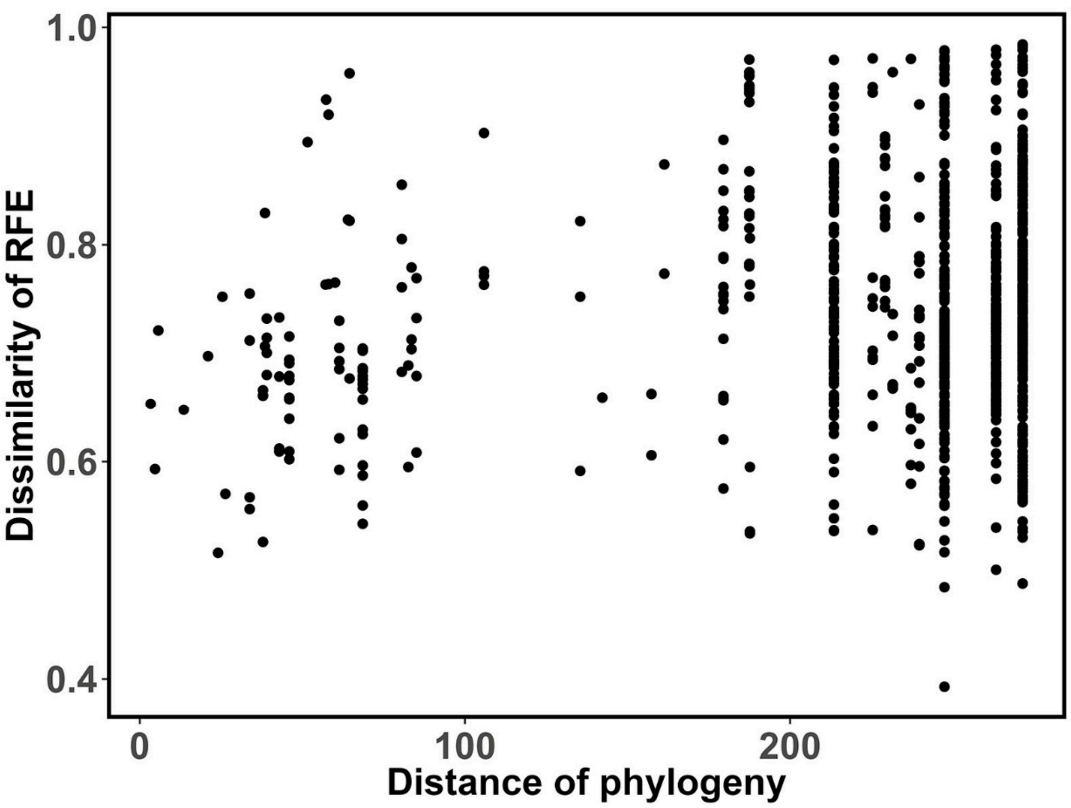
1.0
0.8
0.6
0.4
Dissimilarity of RFE
0
100
200
Distance of phylogeny

Figure S3

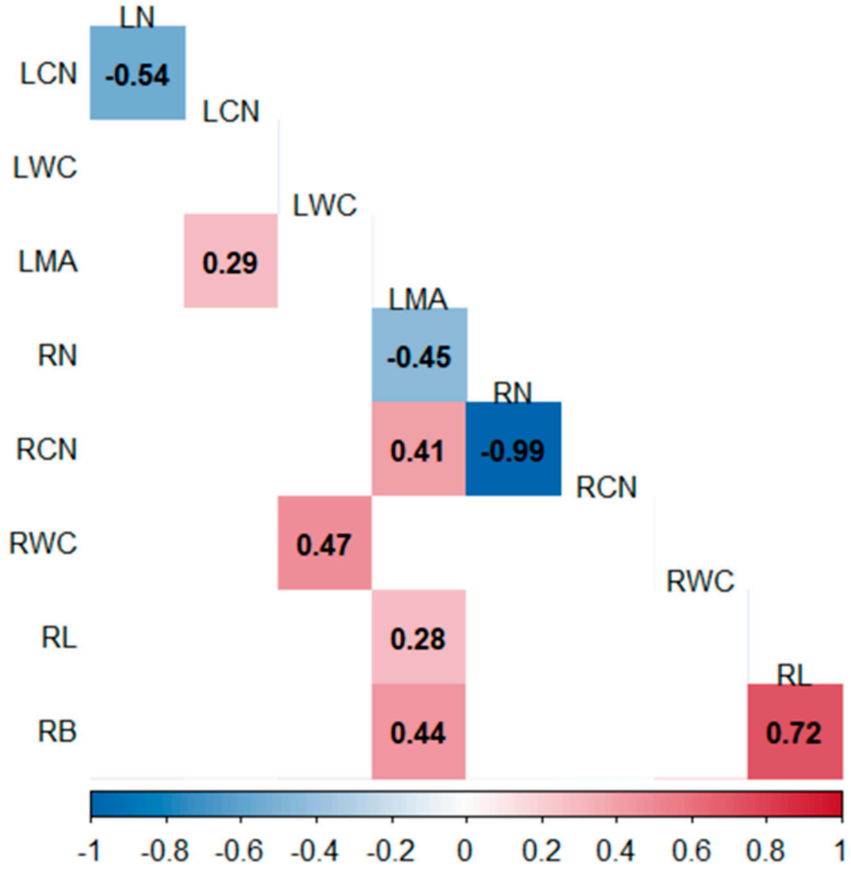
LN
LCN
LWC
LMA
RN
RCN
RWC
RL
RB
-0.54
0.29
-0.45
0.41
-0.99
0.47
0.28
0.44
0.72
-1
-0.8
-0.6
-0.4
-0.2
0
0.2
0.4
0.6
0.8
1

Figure S4

Table S1

Family	Genus	Species
Gentianaceae	*Gentiana*	*Gentiana abaensis*
Ranunculaceae	*Trollius*	*Trollius farreri*
Ranunculaceae	*Anemone*	*Anemone rivularis*
Asteraceae	*Taraxacum*	*Taraxacum lugubre*
Poaceae	*Elymus*	*Elymus nutans*
Poaceae	*Koeleria*	*Koeleria macrantha*
Apiaceae	*Tongoloa*	*Tongoloa elata*
Asteraceae	*Anaphalis*	*Anaphalis flavescens*
Apiaceae	*Angelica*	*Angelica sinensis*
Asteraceae	*Saussurea*	*Saussurea nigrescens*
Rosaceae	*Argentina*	*Argentina anserina*
Rosaceae	*Sibbaldianthe*	*Sibbaldianthe bifurca*
Poaceae	*Deschampsia*	*Deschampsia cespitosa*
Rosaceae	*Potentilla*	*Potentilla discolor*
Fabaceae	*Tibetia*	*Tibetia himalaica*
Amaryllidaceae	*Allium*	*Allium sikkimense*
Asteraceae	*Aster*	*Aster alpinus*
Fabaceae	*Oxytropis*	*Oxytropis ochrocephala*
Asteraceae	*Ligularia*	*Ligularia virgaurea*
Asteraceae	*Leontopodium*	*Leontopodium leontopodioides*
Poaceae	*Agrostis*	*Agrostis hugoniana*
Rubiaceae	*Galium*	*Galium spurium*
Ranunculaceae	*Delphinium*	*Delphinium caeruleum*
Geraniaceae	*Geranium*	*Geranium wilfordii*
Asteraceae	*Artemisia*	*Artemisia frigida*
Lamiaceae	*Scutellaria*	*Scutellaria hypericifolia*
Plantaginaceae	*Veronica*	*Veronica eriogyne*
Lamiaceae	*Ajuga*	*Ajuga ovalifolia*
Gentianaceae	*Gentiana*	*Gentiana formosa*
Euphorbiaceae	*Euphorbia*	*Euphorbia esula*
Fabaceae	*Lathyrus*	*Lathyrus quinquenervius*
Ranunculaceae	*Thalictrum*	*Thalictrum pseudoramosum*
Ranunculaceae	*Anemone*	*Anemone coelestina*
Gentianaceae	*Halenia*	*Halenia elliptica*
Fabaceae	*edysarum*	*Hedysarum sikkimense*
Asteraceae	*Ajania*	*Ajania przewalskii*
Poaceae	*Poa*	*Poa pratensis*
Orobanchaceae	*Euphrasia*	*Euphrasia pectinata*

Asteraceae	*Saussurea*	*Saussurea stella*
Orobanchaceae	*Pedicularis*	*Pedicularis anas*
Poaceae	*Festuca*	*Festuca ovina*
Amaryllidaceae	*Allium*	*Allium chrysanthum*
Caryophyllaceae	*Silene*	*Silene gallica*
Iridaceae	*Iris*	*Iris tectorum*
Asteraceae	*Aster*	*Aster diplostephioides*
